# A review of the findings and theories on surface size effects on visual attention

**DOI:** 10.3389/fpsyg.2013.00902

**Published:** 2013-12-09

**Authors:** Anne O. Peschel, Jacob L. Orquin

**Affiliations:** MAPP Centre for Research on Customer Relations in the Food Sector, Department of Business Administration, Aarhus UniversityAarhus, Denmark

**Keywords:** advertising, eye movements, surface size, visual attention, saliency

## Abstract

That surface size has an impact on attention has been well-known in advertising research for almost a century; however, theoretical accounts of this effect have been sparse. To address this issue, we review studies on surface size effects on eye movements in this paper. While most studies find that large objects are more likely to be fixated, receive more fixations, and are fixated faster than small objects, a comprehensive explanation of this effect is still lacking. To bridge the theoretical gap, we relate the findings from this review to three theories of surface size effects suggested in the literature: a linear model based on the assumption of random fixations (Lohse, 1997), a theory of surface size as visual saliency (Pieters etal., 2007), and a theory based on competition for attention (CA; Janiszewski, 1998). We furthermore suggest a fourth model – demand for attention – which we derive from the theory of CA by revising the underlying model assumptions. In order to test the models against each other, we reanalyze data from an eye tracking study investigating surface size and saliency effects on attention. The reanalysis revealed little support for the first three theories while the demand for attention model showed a much better alignment with the data. We conclude that surface size effects may best be explained as an increase in object signal strength which depends on object size, number of objects in the visual scene, and object distance to the center of the scene. Our findings suggest that advertisers should take into account how objects in the visual scene interact in order to optimize attention to, for instance, brands and logos.

## INTRODUCTION

For an advertisement to be effective, the advertised information must invariably capture consumers’ attention, ideally fast and reliable. To meet this end advertisers often enlarge important objects or messages, for instance, by increasing the size of magazine ads and billboards or the size of important elements within the ad. A large object in an advertisement is more likely to attract attention than a small one (Wedel and Pieters, 2007) and using the optimal size of ad elements furthermore can lead to downstream effects such as increased sales (Zhang et al., 2009). While this might be a satisfactory conclusion from a business perspective, there are only few theoretical attempts to explain why surface size increments affect attention. However, an improved understanding of how surface size affects attention would contribute to research on visual perception of advertising as well as practice by allowing a more systematic approach to the study and application of surface size effects.

Our paper aims to bridge this research gap by first summarizing the results of a literature review including studies on surface size effects on eye movements. In a second step we relate the findings to three theories suggested in the literature: a linear model based on the assumption of random fixations (Lohse, 1997), a theory of surface size as a consequence of visual saliency (Pieters et al., 2007), and a theory of surface size based on competition for attention (CA; Janiszewski, 1998). As a final step we propose a fourth model of surface size effects called “demand for attention” based on a revision of the theory of CA. This model improves the model of CA by adjusting the underlying assumptions based on previous research. In the last section we evaluate the models by reanalyzing a large eye tracking dataset from a study on consumer decisions.

The effect of surface size on attention has previously been reviewed by Wedel and Pieters (2006) as well as Orquin and Mueller Loose (2013). Neither of these reviews addressed the theoretical underpinnings of surface size effects.

## LITERATURE SEARCH AND FINDINGS

The Web of Science, Scopus, PsycINFO, and Google Scholar databases were searched using a combination of keywords related to surface size and visual attention. Additional searching was carried out using literature lists and through contact with authors. The literature search revealed 19 studies fulfilling the inclusion criteria of reporting surface size effects on visual attention operationalized by eye movement measures. Most of the studies were conducted using print advertisements, focusing on brand, pictorial, and text elements (Lohse, 1997; Rosbergen et al., 1997; Janiszewski, 1998; Wedel and Pieters, 2000; Rayner et al., 2001, 2008; Pieters et al., 2002, 2007, 2010; Pieters and Wedel, 2004, 2007; Ryu et al., 2009; Zhang et al., 2009; Boerman et al., 2011). Participants were asked to leaf through magazines while their fixations on the target advertisement were recorded by eye tracking equipment.

The most commonly reported fixation measures were fixation likelihood (FL), fixation count (FC), total fixation duration (TFD), and time to first fixation (TTF). FC and TFD are closely related variables indicating the number of fixations on a stimulus and the total duration of all fixations on the stimulus. FL is the estimated probability that an area will capture attention while attention capture itself is a binary variable indicating whether the stimulus was fixated or not. TTF indicates how fast an area is fixated after stimulus onset. An increase in the first three variables is often referred to as an increase in attention while the opposite is true for TTF (for an overview of fixation measures, see Holmqvist et al., 2011).

The findings from the identified studies were grouped according to the dependent variable in question (see **Table 1**). The most commonly reported dependent variables were TFD, followed by FC and FL while TTF was rarely reported. Overall, the studies show that increasing the surface size of an element significantly increases FC, FL, and TFD toward the enlarged object. The studies reporting TTF also suggest that large objects are fixated faster than small objects. The results reveal a strong and robust effect of surface size on attention, i.e., large objects receive more attention, are more efficient in capturing attention, and do so faster than small objects.

**Table 1:** Overview of the existing literature and findings.

Reference	Stimulus	Fixation count	Fixation likelihood	Total fixation duration	Time to first fixation
Nixon (1924)	Magazine ad			+	-
De Graef etal. (1990)	Line drawings			+	
Lohse (1997)	Magazine ad		+	+	-
Rosbergen etal. (1997)	Magazine ad			+/{ø}	
Janiszewski (1998)	Catalog ad			+	
Goldberg etal. (1999)	Nutrition label			{ø}	-/+
Wedel and Pieters (2000)	Magazine ad	+			
Rayner etal. (2001)	Magazine ad	+		+	
Pieters etal. (2002)	Magazine ad	+			
Pieters and Wedel (2004)	Magazine ad		+/{ø}	+/-	
Pieters etal. (2007)	Feature ad		+/{ø}	+/{ø}	
Pieters and Wedel (2007)	Magazine ad		+	+	
Rayner etal. (2008)	Magazine ad	+		+	
Ryu etal. (2009)	Magazine ad	+		+	
Chandon etal. (2009)	Product shelf	+/{ø}	+/{ø}		
Zhang etal. (2009)	Feature ad			+	
Pieters etal. (2010)	Magazine ad			+/{ø}	
Boerman etal. (2011)	Magazine ad		+/-		
Orquin etal. (2012)	Product packages		+		
Peschel etal. (2013)	Product packages		+		

*+, significant positive effect; -, significant negative effect; {ø}, no significant effect.
*

Although the main effects of surface size on attention are relatively clear, the findings on interaction effects with stimulus class are mixed. Several studies have found that the effect of surface size depends on the class of stimulus, such as the brand, pictorial, or text element in an advertisement (Rosbergen et al., 1997; Goldberg et al., 1999; Pieters and Wedel, 2004; Pieters et al., 2007, 2010; Chandon et al., 2009). However, the findings on interaction effects do not reveal a consistent pattern across studies. Pieters and Wedel (2004), for example, found a significant effect of text element surface size on FL and TFD in magazine advertisements but no effect of surface size for brand or pictorial. Contrary to this, in a study on feature advertisements, the effects were reversed with only the text element being non-significant (Pieters et al., 2007). A further contradiction was reported by Pieters et al. (2010) in a study on magazine ads, where again the TFD toward text elements was unaffected by increases in the surface size. Rosbergen et al. (1997) and Goldberg et al. (1999) only found significant effects of surface size on TFD for specific consumer segments. These authors also found that surface size effects on TTF differed between stimulus classes.

Three studies analyzed how increasing the surface size of one element affects attention to other elements, i.e., how elements compete for attention (Goldberg et al., 1999; Pieters and Wedel, 2004; Boerman et al., 2011). These studies found significant negative effects of increasing the surface size of one element on FL, fixation duration, and TTF toward other elements. However, this CA effect was not consistent across studies. Boerman et al. (2011) found that increasing the size of text elements significantly decreases FL toward pictorial elements. Pieters and Wedel (2004), on the other hand, found that increasing the size of text elements significantly decreases the TFD toward brand elements, however, not toward pictorial elements. Furthermore, the study by Goldberg et al. (1999) showed that other distracting elements, such as anchor lines, decreased the time to first fixate on the enlarged version of the target stimulus.

This brief overview shows that despite a strong and robust effect of surface size on attention, a deeper inspection of the findings produces a mixed picture. Understanding the mechanisms behind the effect of surface size on attention could contribute to an explanation of these apparent inconsistencies. In the following section we introduce four theories on surface size effects on attention. We review the assumptions and predictions of the theories and relate them to the findings from the literature review. As a final evaluation of the theories, we reanalyze an eye tracking data set thereby providing a direct comparison of how well the models describe eye movements in a naturalistic consumer choice situation.

## THEORIES OF SURFACE SIZE EFFECTS ON ATTENTION

### RANDOM FIXATIONS

The first and simplest theory of surface size effects proposed by Lohse (1997) states that the size of an object determines the number of random fixations landing on the object. According to this theory, an object covering 1% of the total surface will receive 1% of fixations; an object covering 2% will receive 2% of fixations, etc. Object surface size increments therefore influence attention metrics linearly resulting in a decrease in TTF, and increase in FC, FL, and TFD.

The simplicity of the theory is appealing but it makes a strong assumption about a random distribution of fixations. Although some eye movement theories assume a stochastic distribution of attention (Krajbich et al., 2010), it is unlikely that fixations are randomly distributed across visual scenes. In fact, one could argue that fixations are exactly the opposite of random, i.e., each fixation is computed to maximize information acquisition (Hayhoe and Rothkopf, 2011) which speaks against the fundamental assumptions of this theory.

The reviewed literature suggests that surface size does not influence attention linearly but rather logarithmically. In an early study conducted by Nixon (1924), 30 participants were observed while looking at magazine advertisements which either occupied a whole or half a page. The ratio of TFD of the full relative to the half page advertisements was estimated at 1 to 0.74. This indicates greater attention toward the full page advertisement, however, not in the linear manner as described above, which would predict that reducing object size by 50% would result in a loss of 50% of fixations.

Lohse (1997) conducted a study with 32 participants looking at yellow page advertisements in which participants were asked to find three businesses in each of eight categories. The study showed that the number of fixations per advertisement increased with the size of the advertisement; small advertisements, however, maximized the number of fixations per square inch. In other words, small advertisements gained attention more efficiently than large ones which again contradict the assumption of a random distribution of fixations.

At a simulated supermarket shelf, Chandon et al. (2009) examined the effect of number of product facings on attention. The number of product facings is an indicator of how large an area of the shelf is occupied by a particular brand, i.e., the surface size for that brand. The study revealed a significant positive effect of number of product facings on FC and FL for an increase from four to eight product facings. Adding four more facings resulted in a marginal but significant increase in both attention measures.

Finally, in a study comparing 198 different products Orquin et al. (2012) examined the effect of surface size of individual product packaging elements on attention. The analysis revealed a better model fit when the surface size variable had been log transformed indicating non-linear effects of size on TFD.

The reviewed studies show a very high agreement on non-linear effects of surface size on attention. All studies point to a logarithmic effect of size with the higher gains occurring for small objects and a diminishing marginal effect for large ones. We find no evidence supporting the prediction that surface size has a linear effect on attention.

### SIZE AS SALIENCY

Another interpretation of surface size effects is that size increments lead to higher visual saliency and therefore affect attention through saliency (Pieters et al., 2007). Visual saliency is often manipulated through stimulus contrast or luminance (e.g., Foulsham and Underwood, 2009; Nordfang et al., 2013) but more advanced computational models integrate several feature dimensions to compute stimulus pop-out. These dimensions typically include color, orientation, and contrast. The computational models integrate these feature layers into a saliency map predicting the relative saliency of each pixel in the image analyzed (Itti and Koch, 2001). If size effects are a function of visual saliency, as suggested, it then follows that size effects share psychometric properties with visual saliency such as shorter TTF and higher FL for more salient objects (Itti and Koch, 2001). Another property of saliency is that the effect is easily disrupted by task instructions (Einhäuser et al., 2008), semantic or contextual cues about a visual scene, feature-based attention, object representations, and rewards for task performance (Kowler, 2011). According to the size as saliency theory, the same should be true for surface size effects. Another important consequence of the theory is that if size is a function of saliency, then the effect size of surface size should be in the same range as the effect size of saliency, but never exceeding it. In line with this, when controlling for visual saliency, size effects should be minimal or non-existent.

Orquin et al. (2012) studied the effect of size and saliency of product packaging elements on FL. Spearman’s Rho correlation between size and saliency of each packaging element were considerably strong (ρ_s_ = 0.434). The correlation coefficient therefore suggests that at least some of the effect of size on attention is due to increments in saliency.

Pieters and Wedel (2007) studied gaze duration to brand, pictorial, headline, and body text elements of advertisements under various viewing goals (ad memorization, ad appreciation, brand learning, or brand evaluation). The study revealed that surface size had a significant positive effect on attention independent of processing goals indicating that, unlike saliency, surface size effects were not disrupted or affected by task instructions.

Regarding the statement that surface size effects cannot exceed saliency effects, several studies suggest otherwise. In the previously described study by Lohse (1997), advertisements were distinguished by being black and white or containing some red color. In a layout of black and white posting, the red advertisement is considered more salient than the others due to increased contrast. Accordingly, these advertisements received more attention in terms of increased FL, TFD and decreased TTF. However, size effects were stronger than saliency effects for all three measures.

In the Pieters et al. (2007) study saliency was operationalized by assessing target distinctiveness and distractor heterogeneity. An element with high target distinctiveness stands out relative to the distractor item due to its size and orientation. High distractor heterogeneity is characterized by distractors of different size, shape, and orientation. Both high target distinctiveness and low distractor heterogeneity result in higher target saliency. The results showed a significant influence of both target distinctiveness and distractor heterogeneity on FL and a significant effect of target distinctiveness on TFD. Yet, as observed before, the effect of surface size was much stronger than the target distinctiveness and distractor heterogeneity measures.

Brand identifiability served as a measure of saliency in the study by Pieters et al. (2010). This measure incorporated different features such as contrast and heterogeneity of background elements. Brand identifiability had no significant effect on attention toward advertisement elements or toward the advertisement as a whole. Contrary to this, significant surface size effects were observed for individual elements.

Similar results were obtained by Peschel et al. (2013) where size and saliency were manipulated in an orthogonal design. Saliency did not affect attention significantly; size on the other hand influenced FL significantly. Since the study manipulated visual saliency and surface size in an orthogonal design, we can conclude that surface size had a significant effect independent of the level of saliency. In Orquin et al. (2012) both size and saliency showed a significant effect on FL but the influence of surface size on attention was twice as strong as that of saliency.

Overall, the theory of size as saliency can only be accepted in parts. There are sound theoretical reasons and empirical evidence that support the assumption of a substantial correlation between size and saliency. However, the effects of surface size on attention cannot be subsumed to visual saliency as the effect is both independent of and stronger than that of visual saliency.

### COMPETITION FOR ATTENTION

The theory of surface size referred to here as “competition for attention” is derived from work by Janiszewski (1998). It is based on the assumption that object-based signal strength measured by visual receptor cones deteriorates as a function of distance from the focal point. This is due to the fact that less receptor cones are located outside the fovea which the center of the visual image is projected onto. Peripheral objects are therefore projected onto an area with fewer receptors, resulting in a weaker signal strength. However, increasing the size of peripheral objects leads to projection on a larger area of receptors resulting in measurably stronger signal strength. The theory further assumes that based on their attentional demand, objects in a visual scene compete for attention. Attentional demand refers to an object’s strength to attract attention based on its size and the distance to the object which is currently fixated. The theory further states that the CA, when fixating one object, is equal to the sum of attentional demand from surrounding objects. Objects with a low surrounding CA will attract more fixations. Janiszewski (1998) calculated an object *i*’s CA as the sum of the size to distance ratio of all surrounding objects *j* which we will refer to as CAJA**_i_:

(1) $$C\,A\,J\,A_{i}=\sum_{1}^{j}\frac{S_{j}}{D_{i-j}}.$$

Where *S*_j_ refers to the size (in degrees) of the surrounding objects in question and *D*_i__-_**_j_ is the distance (in degrees) from object *j* to object *i.* In his first study, Janiszewski (1998) found a significant negative correlation of CAJA**_i_ with an average gaze time (*r* = -0.46) indicating that an object faced with a lot of CA attracted less fixations. In an additional study he found that incorporating CAJA**_i_ in a regression model next to size as a factor explained significantly more of the observed variance.

**FIGURE 1:**
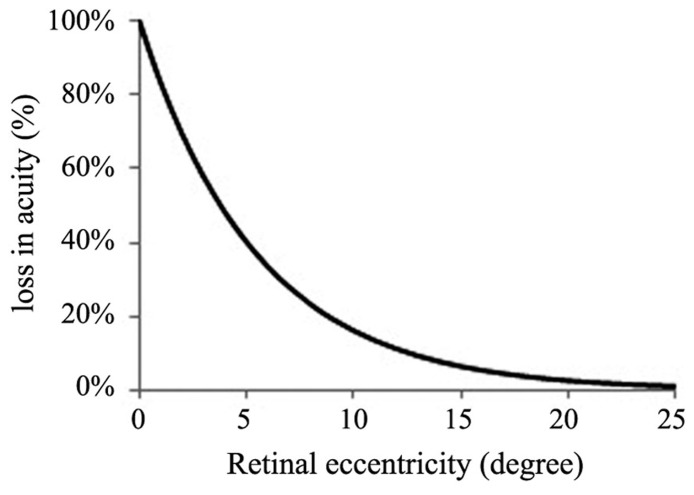
**Loss of visual acuity as a function of degrees of retinal eccentricity**.

While deriving his model, Janiszewski (1998) pointed out that the attentional demand of any object is equal to its size discounted for loss of acuity. We incorporate this idea into his model below. The loss of acuity is a consequence of diminishing visual acuity in retinal eccentricity and it has been shown that in order to maintain visual acuity an object must increase by 0.2° in size for each degree of retinal eccentricity (Anstis, 1974). To maintain acuity as an object recedes from the currently fixated location, it must grow by 0.2°. If it, on the other hand, maintains its current size it follows that acuity is reduced by a factor of 1/1.2 or 83.33% for each degree of retinal eccentricity (Anstis, 1974). The acuity loss function is illustrated in **Figure 1**. It documents the loss of acuity in percent for each degree increase of retinal eccentricity.

To compute the acuity of an object of size *S* and distance *D* from a current fixation, the following formula can be applied:

(2) $$A\,c\,u\,i\,t\,y\,\left(S,D\right)=S*0,833^{D}\,.$$

Incorporating visual acuity loss in the computation of CA for object *i* (CA**_i_) with *j* surrounding objects results in the following formula:

(3) $$CA_{i}=\overset{j}{\underset{1}{\sum }}S_{j}*0,\text{​}833^{D_{i-j}}.$$

Here each object *j* is discounted for acuity loss seen from the fixation position of object *i*. The idea is that when fixating an object *i*, all surrounding objects are competing to draw attention away from the object. Depending on their distance and size, the surrounding objects will impose an either greater or weaker CA.

Since we are interested in explaining size effects on attention and not the competition of attention caused by these effects, we refine the model further. Considering the overall CA in the visual scene, the sum of relative CA imposed on any object can be computed as the proportion of CA (CA**_i_) for that object relative to the overall sum of $C\,A\,\left(\sum_{j}^{N}C\,A_{j}\right)$ in the visual scene. To further derive the relative measure of attention directed to an object *i* (*A*_i_), the proportion of the inverse of CA serves as our model of demand for attention based on CA:

(4) $$A_{i}=\frac{1/C\,A_{i}}{\sum_{j}^{N}1/C\,A_{j}}\,.$$

**FIGURE 2:**
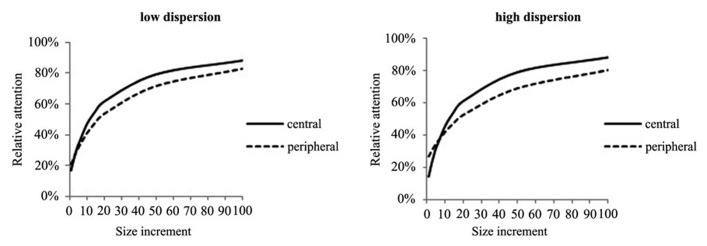
**Effects of size on attention for central and peripheral objects under low and high dispersion of objects**.

This model predicts that, everything else being equal, the more objects there are in a visual scene the less attention will be directed to any object. In addition, it can be derived that the effect of increasing object size on attention is stronger for smaller set sizes, i.e., visual scenes containing fewer objects. If all objects have the same size and distance to each other, each object will receive 1/N measures of attention. However, with increasing size and various degrees of dispersion, differences in relative attention devoted to central or peripheral objects can be observed, as illustrated in **Figure 2**. The *x*-axis describes size increments of a peripheral or central object by a factor of 10. In the low dispersion condition, peripheral objects are defined to be equally dispersed around the center with a distance factor of 5. In the high dispersion condition, the peripheral object is located twice as much outside the center. According to the model, an object which is located on the periphery relative to the other objects in the visual scene should initially receive more visual attention due to less CA. However, size increments gains are lower compared to an object which is located closer to the center. As opposed to the two theories reviewed above, the theory of competition of attention predicts that increasing object size has diminishing marginal effect on attention.

Evidence in favor of the theory of CA is scarce as the identified papers do not provide information about set size or position of the stimuli. One prediction, however, is confirmed as diminishing marginal effects of surface size on attention have been extensively covered in the previous section. To further assess the robustness of the model on a theoretical level, we proceed to discuss the assumptions in more detail. The theory is based on three main assumptions: first of all, in order to compute CA it is assumed that all objects are fixated, recall that CA is the sum of demand for attention of all surrounding objects. Second the theory assumes that there is no effect of the object centrality in the visual scene, i.e., objects that are positioned more centrally in the visual scene are not fixated more often than peripheral ones. The third and most important assumption is that an object’s signal strength is a function of its visual acuity.

Regarding the assumption of all objects being fixated, it is clear that this can only be the case under certain conditions such as when there is a limited number of objects in the visual scene. It has, for instance, been demonstrated that increasing set size leads to non-attendance, i.e., objects not being fixated, and that increasing the set size further leads to increasing non-attendance (Orquin and Mueller Loose, 2013). For a general theory of surface size it is therefore not appropriate to assume that all objects are fixated. The second assumption that there is no effect of centrality is also difficult to sustain as a general principle as it has been shown repeatedly that participants tend to gaze at the middle of a visual scene (Vincent et al., 2009; Tatler et al., 2011).

The third assumption about object signal strength as a function of visual acuity is more difficult to evaluate. To understand the claim about object-based signal strength, note should be made of the fact that objects have been shown to predict attention better than, for instance, visual saliency (Scholl, 2001; Einhäuser et al., 2008). A number of computational models have been developed to explain object-based attention (Walther and Koch, 2006; Orabona et al., 2008). The tenet of these models is that visual selection occurs after the perception of an object (Rensink, 2000) or even after categorization of the object (Bundesen, 1998). These findings suggest a strong effect of object-based attention in the sense that identifying or recognizing something as an object increases the likelihood of fixating it.

If increasing object surface size augments object identification, it should also have an effect on attention. It has already been shown that size is a strong predictor of visual acuity, which determines how well a stimulus is detected and identified (Anstis, 1974; Strasburger and Rentschler, 1996; Kondo et al., 2008). However, most of the studies on object identification in eccentricity looked at isolated objects, which is not ecologically valid, as visual scenes in general contain several objects. In addition, the number of objects in a scene was shown to create visual clutter (Rosenholtz et al., 2007) or visual crowding (Levi, 2008; Whitney and Levi, 2011), which prevents the identification of target objects. Nevertheless, it has been argued that crowding is independent of object size (van den Berg et al., 2007) which would mean that clutter and crowding can be ignored when trying to describe effects of object size.

However, a few studies indicate that crowding is not size independent but that differences in size and shape between target and flankers diminish crowding effects (Treisman and Gelade, 1980; Nazir, 1992; Kooi et al., 1994; Levi and Carney, 2009). Results of these studies showed increased target identification when flankers were larger than the target, i.e., small object size led to increased identification when the target was increasingly distinct from the flankers. This suggests that surface size can play a larger role than merely determining acuity. Whether flanker effects of this type occur in natural vision is difficult to say as most experiments on crowding used highly controlled lab experiments. What seems clear is, however, that surface size does affect an object’s signal strength and that this signal is furthermore dependent on the distance of the object to the location currently fixated. This speaks in favor of the third assumption stating that an object’s signal strength is a function of its visual acuity.

### DEMAND FOR ATTENTION

Taking the above considerations into account, we propose an alternative model to the theory of CA (Janiszewski, 1998). The assumption concerning object-based attention was found plausible as described above. However, the computation of CA**_i_ (Eq. 3) faces two theoretical problems as it is based on the assumption of all objects being fixated as well as the assumption that object centrality plays no role on attention. To address these challenges we propose a revision of the model of CA. Our model is based on the demand for attention of an object *i* (DA**_i_) based on its signal strength relative to all other objects in the visual scene. To address the centrality issue we propose that demand for attention is computed as an object’s visual acuity (Eq. 2) as seen from the center of the visual scene (D_c_). In order to assess an object’s relative demand for attention, thus incorporating CA in this model, the proportion of demand for one object is divided by the total demand for attention in the visual scene:

(5) $$DA_{i}=\frac{S_{i}*0,\text{​}833^{D_{c}}}{\sum_{1}^{j}S_{j}*0,\text{​}8333^{D_{c}}}.$$

**FIGURE 3:**
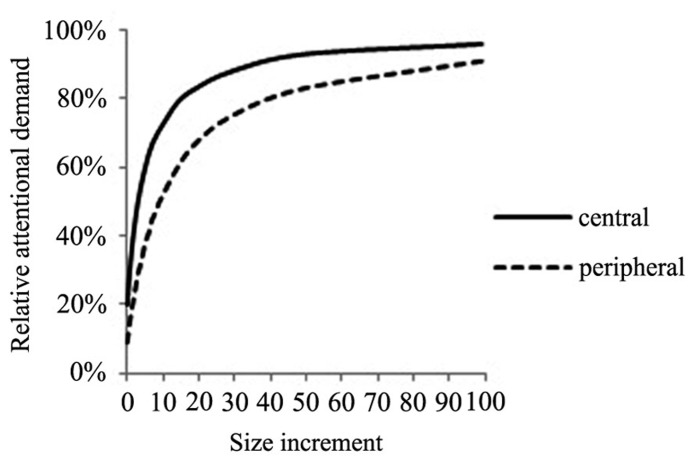
**Effect of size increments on relative attentional demand for a centrally and peripherally located object**.

The model predicts that objects with a higher relative demand for attention are fixated earlier and with a higher likelihood. If all objects have the same signal strength, each object will receive 1/*N* amounts of relative attention. This means that, holding signal strength constant, increasing the number of objects in the visual scene will reduce the relative amount of attention per object as a monotonic function of the set size. Plotting the effects of size increments on attention shows that central objects demand most attention initially and also gain more from size increments than peripheral objects as illustrated in **Figure 3**. Again the *x*-axis describes size increments of a peripheral or central object by a factor of 10. The peripheral object in focus is located away from the center by a distance factor of 10. Similar to the model of CA, the model predicts diminishing marginal effects of surface size on attention.

## EMPIRICAL EVALUATION OF THE THEORIES

In order to evaluate the theoretical models derived above, we reanalyzed data from Orquin et al. (2012). FL, TFD, and TTF from 123 Danish consumers were analyzed. The total stimulus sample contained 198 product images from four food product categories (yogurt, milk, cheese, and butter). For each product, seven areas of interest were defined (brand, category, fat percentage, organic label, keyhole label, GDA label, pictorial). Each product had on average six areas of interest. Surface size, saliency, and position on the packaging were determined for each element. The saliency measure was obtained using a saliency algorithm developed by Itti and Koch (2001). The experimental stimuli were existing market products which might restrict the co-occurrence of some features. The data was collected using a Tobii 2150 eye tracker and each product image was displayed on the screen approximating the natural size of the product. The participants were asked to choose a product from a choice set of four products. Each product was viewed separately and for as long as the participants needed and the decision was made only after having viewed all four products. Although product packaging is different from advertising, the cognitive processes that guide eye movements should be comparable. We therefore argue that it is reasonable to transfer findings from product packaging to other areas such as advertisement research.

According to the formulas introduced above, CA as used by Janiszewski (1998) (CAJA), relative attention based on CA including (*A*_i_), demand for attention excluding visual acuity loss (DA_no___acuity_) and demand for attention (DA**_i_) were derived. Based on that, Spearman’s Rho pairwise correlations of CAJA, A**_i_, DA_no_acuity_ and DA**_i_ with aggregated attention measures (FC, FL, TFD, and TTF) were performed to identify the strength of association of the models using empirical data. We chose correlations as a measure of association between the two variables because it is straightforward in interpretation and constitutes an appropriate measure of effect size. In order to compare the performance of the models with other measures, size, distance to the center, and saliency, values were also correlated with the fixation data.

The results displayed in **Table 2** demonstrate that demand for attention showed the strongest relationship with all fixation measures. All correlations were between 0.5 and 0.6, indicating a strong relationship between the measure of demand for attention and fixation data. Correlations of size were similar to demand for attention, yet slightly less pronounced suggesting that our model of demand for attention contributes to explain size effects on attention. Distance to center showed significant correlations with all attention measures as well, even though less pronounced than size and demand for attention. To ensure the contribution of visual acuity loss, we also calculated demand for attention without acuity loss (DA_no_acuity_). This measure still performed better than the other CA measures but resulted in considerably weaker correlations than DA**_i_. This means that adding distance to the center and visual acuity loss to the model contributes to the explanation of size effects.

**Table 2:** Pairwise correlations of potential predictors and fixation measures.

Variable	Mean	Mean	Mean	Mean
	FC	FL	TFD	TTF
Demand for attention (DA**_i_)	0.593	0.594	0.555	-0.498
Surface size	0.564	0.581	0.511	-0.422
Demand for attention (DA_no_acuity_)	0.465	0.388	0.429	-0.395
Distance to center	-0.366	-0.462	-0.371	0.363
Saliency	0.209	0.306	0.192	-0.162
Attention based on CA**_i_ (*A*_i_)	0.123	0.012	0.088	-0.029
CA**_i_ Janiszewski	-0.051	0.084	-0.037	n.s.

*All Spearman’s Rho (ρ_s_) correlations are significant at *p* < 0.01 level unless labeled n.s.*

Saliency was considerably less correlated with attention data but indicated that more salient objects receive more attention in terms of all fixation measures. *A*_i_ was weakly but significantly correlated with all fixation measures and all correlations point in the expected direction. Nevertheless, this model did not proof useful when trying to explain size effects on attention, because size as a measure on its own was stronger correlated with fixation data. The model introduced by Janiszewski (1998) was even weaker correlated with the empirical data. In addition, the correlation between CA and FL pointed in the wrong direction and the correlation with TTF was not significant. On top of that, we could not find a correlation of CA and TFD as strong as reported in Janiszewski’s (1998) study.

The results of this section suggest that the models of CA did not contribute to explaining size effect on attention. Contrary, the model of DA**_i_ was closest to our empirical fixation data. The finding supports the assumption of a central fixation bias and the necessity to account for visual acuity loss when analyzing surface size effects on visual attention.

## DISCUSSION

Overall the reviewed studies showed that surface size had a significant positive effect on FC, FL, and TFD; however, magnitude differed in between objects and context of the stimuli. In order to get a more profound understanding of size effects and to classify their impact on attention, four theories of surface size effects on attention were evaluated in this paper. The first theory, explaining surface size effects with a linear increase in attention due to random fixations, could be rejected based on ample evidence showing that surface size effects on attention are logarithmic (Lohse, 1997). Small objects gained more from size increases than large objects (Chandon et al., 2009) suggesting that size increments were limited in the capacity to increase attention. All in all, these findings explain that size increments do not increase the probability that fixations randomly land on a larger area. Since small elements gain more from size increments, a logarithmic distribution of gains in attention is reasonable. Consequently size effects do not influence attention linearly based on random fixations but must be explainable with more targeted information acquisition.

Size as function of saliency comes closer to explaining the effect of increased attention based on size increments. Objects that are salient attract attention and suggest a greater interest to the observer in free viewing tasks (Parkhurst et al., 2002). However, this effect diminishes when applied to real-world search tasks (Henderson et al., 2007). Since most of the reviewed studies were conducted under free viewing conditions, it seems reasonable to expect an effect of visual saliency on attention. Based on the theory presented, surface size is seen as a dimension of saliency (Pieters et al., 2007). This would mean that an object’s size increments affect attention because the object becomes more salient but not due to the size increment in itself. Indeed, a correlation exists between size and saliency. Nonetheless, the results presented show that size cannot be seen as a property of saliency but that it is a measure on its own showing consistently stronger effect sizes than saliency. Interestingly though, the correlation of saliency with fixation data was stronger than that of CA. This confirms the potential of saliency to attract attention, yet to a lesser extent than size. This is supported by the significant interaction effect of size and saliency found in Orquin et al. (2012). It is reasonable to assume that a salient target will gain more attention from size increments than a non-salient one; however, the driver for this effect remains surface size in itself. The theory of size as function of saliency therefore is not supported by our findings.

The models of CA incorporate set size and distance features of surrounding objects in order to explain the effect of size increments on attention. Janiszewski’s (1998) model predicts that an object that is faced with a high degree in CA will receive less attention. Our data revealed weak or non-significant relations going in the opposite direction than that predicted by the model. The data clearly shows that other models are better suited to account for the observations.

The model of attentional demand (*A*_i_) refines the model of CA as it accounts for object signal strength as a function of visual acuity loss relative to the fixated object. In addition, this model delivers clear predictions as to the measure of attention that each object will receive relative to all other objects in the visual scene. This also means that the more objects there are in a visual scene, the less attention each object receives. Furthermore, an increase in size of the fixated object will result in increased attention for this object relative to the competition of attention imposed from all other objects. As described above, the increase in attention is predicted to be shaped as a logarithmic function. The correlations with our attention data were significant and pointed in the expected direction. However, size as a measure on its own was stronger correlated with attention data. Consequently, we do not find evidence supporting that this model provides an explanation of size effects on attention.

As discussed before, the underlying assumptions of the models of CA were not robust when related to findings from the literature. A closer relationship with the data was expected when the assumption of equal distribution of attention in the visual scene was replaced by the assumption of a central fixation bias. The model of demand for attention (DA**_i_) is much simpler than the models of CA as it only consists of the target object’s size and distance to the center relative to the sum of demand for attention from the other objects in the visual scene. Increasing the target object’s size was predicted to result in a logarithmic increase of attention toward that object. Objects which were closer to the center would gain more from size increments than others. The correlations between our fixation data and demand for attention resulted in substantially stronger correlations than all other measures. Consequently, the model of demand for attention performed better than pure surface size measures. This supports the idea that size effects depend on other factors as well such as set size and position. Our model of demand for attention predicts that fixations are equally distributed when objects are equal in size and distance. However, increasing the size of one object, leaving distances equal, should result in more fixations for the larger object. This prediction is in line with previously mentioned findings, namely that crowding effects are dependent on size effects (Treisman and Gelade, 1980; Nazir, 1992; Kooi et al., 1994; Levi and Carney, 2009). In support of our model, demand for attention without visual acuity loss (DA_no_acuity_) performed better than the other CA measures, saliency and distance to the center. Still, this measure was considerably weaker correlated with fixation measures than DA**_i_ and surface size. A potential explanation for the robust performance of DA_no_acuity_ could be the strong effect of the central bias assumption since it is the major difference between demand for attention and the CA models. If this is true, then measuring distance from the target object to the center and to all other objects is one of the major features that need to be taken into account when explaining size effects.

Based on our findings, size effects on attention can be explained by an object’s signal strength, which is a function of visual acuity loss and distance to the center. Increased signal strength serves as a proxy for visual attention. When observing a visual scene, the center will be the focal point of attention. Surrounding objects compete for attention with less signal strength the further they are away from the center. Increasing object size according to the acuity loss function, however, will compensate for the distance to the center and enhance visual perception of peripheral stimuli. The theoretical model assumes that shifting the position of a stimulus closer to the center, results in an increase in signal strength but to a lesser degree than increasing size. Transferring these results to visual advertising research, an improved layout design might be achieved by systematically organizing the important information based on the signal strength of each element and taking into account that size and position of all advertisement elements influence each other in terms of how much attention each object will gain. Increasing the size of one object increases its signal strength but imposes CA on other elements. Being located as close to the center as possible enhances signal strength but is also dependent on the location and size of other objects in an advertisement. The model of demand for attention, which we suggest, could serve as a systematic approximation to optimize advertising layout in practice; this might improve sales through more efficient attention allocation.

Implementing object signal strength as a function of visual acuity loss and distance to the center is a refinement of the model of CA (Janiszewski, 1998) which contributes to an understanding of surface size effects on attention. Nevertheless, the magnitude of correlations showed that our model can be further improved by other factors that contribute to explain size effects on attention. Overall it can be concluded that size effects on attention depend on their surroundings and can be more effectively predicted when visual acuity loss and distance to the center are accounted for in the model.

Future research should attempt to improve the integration of set size in the model of demand for attention. The current model accounts for set size by incorporating the total demand for attention in the estimation of relative attention to each individual object. However, the decisive factor for relative attention allocated to each object depends on the signal strength of the object. This means that in a visual scene with high heterogeneity in demand for attention, e.g., the target object has high demand for attention and the distractor objects have low demand for attention, the number of surrounding objects should have little or no effect on the amount of relative attention to the target. The question is whether this assumption is realistic or whether there is a minimum influence of the set size on demand for attention.

## AUTHOR CONTRIBUTIONS

The authors contributed in equal shares to this article.

## Conflict of Interest Statement

The authors declare that the research was conducted in the absence of any commercial or financial relationships that could be construed as a potential conflict of interest.
